# Thiourea-formaldehyde-Functionalized Graphene Oxide for the Selective Removal of Copper from Multielement Solution

**DOI:** 10.1021/acsomega.5c13435

**Published:** 2026-04-01

**Authors:** Nicole Ferreira, Thainara Viana, Gil Gonçalves, Cláudia Nunes, Eduarda Pereira, Bruno Henriques

**Affiliations:** † LAQV-REQUIMTE - Associated Laboratory for Green Chemistry, Department of Chemistry, 450630 University of Aveiro, 3810-193 Aveiro, Portugal; ‡ CICECO - Aveiro Institute of Materials, Department of Chemistry, 56062 University of Aveiro, 3810-193 Aveiro, Portugal; § TEMA - Centre for Mechanical Technology and Automation, Mechanical Engineering Department, 56062 University of Aveiro, 3810-193 Aveiro, Portugal; ∥ CICECO - Aveiro Institute of Materials, Department of Materials and Ceramics Engineering, 201876 University of Aveiro, 3810-193 Aveiro, Portugal

## Abstract

Copper (Cu), a critical raw material (CRMs) essential for renewable energy technologies, requires sustainable recovery strategies to ensure supply security and minimize environmental impacts. Recent market trends highlight the growing economic and strategic importance of Cu, reinforcing the need for selective recovery from secondary and waste-derived resources. However, Cu separation from complex waste streams, such as wind turbine leachates, remains challenging due to competition from base metals and rare earth elements. In this study, a thiourea-formaldehyde functionalized graphene oxide (G3DTF) is introduced as a new sulfur- and nitrogen-rich hybrid sorbent for selective Cu recovery under competitive conditions. Unlike conventional graphene-based materials, G3DTF incorporates tailored S/N donor sites within a graphene oxide framework, creating preferential coordination environments for Cu. The sorbent was evaluated in both monoelement and equimolar multielement solutions (100 μM of each element: boron, cobalt, copper, nickel, praseodymium, neodymium, gadolinium, dysprosium). Under optimized conditions, G3DTF achieved up to ≈99% Cu removal while exhibiting negligible uptake of competing ions. Langmuir isotherm analysis yielded a maximum adsorption capacity (*q*_max_) of 23.4 mg g^–1^, indicating strong affinity to Cu. Kinetic analysis revealed condition-dependent behavior. Copper adsorption followed pseudo-first-order kinetics in monoelement systems, whereas chemisorption became rate-limiting under competitive multielement matrices. Response surface optimization identified sorbent dosage as the dominant operational parameter, with optimal performance at 1.1 g L^–1^ and pH 5.2, independent of salinity. XPS analysis confirmed that Cu–S governs selective binding. The performance of G3DTF in real leachate-mimicking systems further validated its selectivity and application potential. These results demonstrate the ability of G3DTF as a sustainable platform for selective Cu recovery from CRM-rich waste streams.

## Graphical abstract

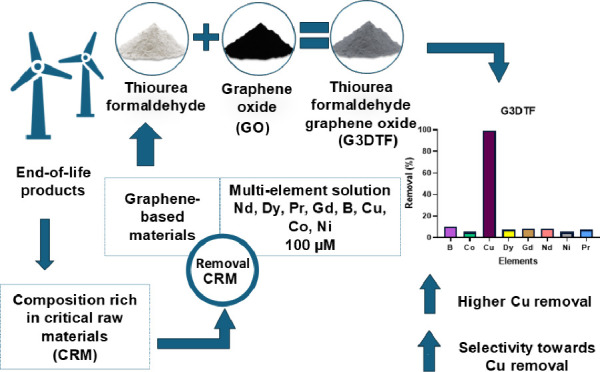

## 1 Introduction

Copper (Cu) is regarded as a key mineral, broadly utilized in the advancement of environmentally friendly technologies and renewable energy production. A distinctive combination of properties, including excellent conductivity (both electrical and thermal), as well as elevated corrosion resistance, makes Cu suitable for a wide range of applications.^1^ Although renewable energy systems are more environmentally friendly and produce fewer greenhouse gas (GHG) emissions, they generally require more Cu than current energy technologies.^2,3^ The International Copper Association shows that Cu use for green energy is likely to rise from 0.8 to 6.7 Mt by 2040 (+11% for electric vehicles, +19% for grid expansion, +7% for renewable energies, compared to +0.5% for traditional energy).^4^ Global Cu demand has increased more than 2-fold over the last four decades and is expected to continue increasing in the coming decades.^2,5^

Between 60 and 90% of the total energy needed for producing primary Cu arises from mining and mineral processing phases, which contribute significantly to various environmental impacts, depending on the ore grade,^5,6^ such as the generation of slags, dust, and aerosols as byproducts that are contaminated with metal­(loids)­s, including potentially toxic ones such as As, Cd, Hg, and Pb.^7^ As Cu deposits become depleted, the specific extraction energy increases. Consequently, a wind turbine’s energy return on investment in 2050 would be 15% lower compared to in 2012.^8^ Therefore, achieving the targeted reductions in GHG emissions will be challenging unless there is a reliable shift toward renewable energy.

The recent Renewable Energy Directive (Directive (EU) 2018/2001) aims for Europe to meet a minimum of 32% of its total energy consumption to be renewable energy by 2030, with wind power making a significant contribution.^9,10^ By 2017, wind power had surged to the second-largest electricity source and was forecasted to meet 25% of Europe’s needs by 2050.^11^ Projections indicate approximately 270,000 wind turbines operating worldwide, taking into account a usual yield of around 3 megawatts (MW) per wind turbine (industrial onshore).^12^ Furthermore, the requirement of Cu for offshore wind energy installations is around 10.5 tons of Cu per MW, whereas onshore wind farms require just 3.9 tons of Cu per MW.^1^

Although researchers differ on whether the world will face increasing Cu shortages in the coming decades,^13^ they collectively emphasize the importance of Cu recycling in ensuring a cleaner long-term Cu supply and reducing environmental impacts.^2^

In 2023, Cu was among the most Critical Raw Materials (CRMs) for the European Union, despite not meeting CRMs thresholds, highlighting its scarcity, high economic value, and indispensability in key sectors of the global economy.^14^ Based on London Metal Exchange data from November 7–10, 2025, the prices of Cu increased by approximately 0.6–0.7%, reaching a range of $10,739–$11,114 USD/tonne following an all-time high near $11,114 USD/tonne at the end of October 2025.^15^ This reflects an overall upward momentum in Cu pricing compared with Q2 2025. This price escalation of Cu highlights the growing strategic importance and reinforces the demand for sustainable strategies to ensure supply security. The recently adopted Critical Raw Materials Act aims to strengthen each stage of the European CRMs value chain, promote circular economy and sustainability, improve the collection of CRM-rich waste (i.e., Waste Electric and Electronic Equipment (WEEE)) and their recycling, and ensure a secondary source of CRMs in the EU market.^16^

Although the Cu recycling input rate at the end of its service life is higher than that of most of the other CRMs (∼55%), it is far from its potential. If WEEE is not rightfully disposed of, large amounts of CRMs can be leached and enter aquatic environments, disrupting the balance of natural ecosystems and threatening human health.

Given the importance of Cu in the technology industry, several conventional separation processes have been employed to remove it from aqueous solutions. These processes include precipitation,^17^ ion exchange,^18^ electrochemical techniques,^19^ solvent extraction,^20^ and membrane filtration.^17^ In recent years, sorption has gained considerable interest as a promising route to remove and recover Cu from solutions.^21^ It is considered one of the most effective and extensively studied approaches in water remediation and hydrometallurgical processes. Among materials investigated for sorption purposes, graphene-based materials, including graphene oxide, have demonstrated significant potential in removing metal contaminants from water.^22,23^ Graphene oxide (GO) has attracted noteworthy interest because it has properties similar to pure graphene while being simpler and cheaper to produce.^24^ It has a large surface area that improves sorption and significant oxygen content (i.e., hydroxyl, carbonyl, epoxy functional groups), making it suitable for functionalization with inorganic and organic chemical structures.^25^ This is particularly advantageous in water remediation applications. A study using Fe_3_O_4_@C/GO nanocomposites for Cu­(II) removal from ultrapure water showed high efficiency (77 mg g^–1^).^26^ Furthermore, a chitosan Schiff base–magnetite–graphene oxide nanocomposite was used to remove Cu from solutions, reaching high Cu removal (111 mg g^–1^).^27^ However, the use of materials based on graphene to remove Cu has only been studied in monoelement solutions of the element or in mixtures with few potentially toxic elements, such as Cd and Pb, offering limited relevance to real waste streams. Complex mixtures containing different metals, such as base metals and rare earth elements (REE), or the selective removal of Cu have never been the focus of research. Moreover, adsorption mechanisms are often generalized without considering multicomponent effects.

The Pearson principle states that Cu­(I) and Cu­(II) have a higher affinity for sulfur and nitrogen atoms, respectively.^28^ Thus, a material containing both thiol and amine functional groups can be beneficial for selectively removing Cu from a multielement solution. Here, we report a thiourea-formaldehyde-functionalized graphene oxide (G3DTF) as a sulfur- and nitrogen-rich hybrid sorbent specifically engineered for selective Cu recovery from complex, multielement systems relevant to wind turbine recycling.^29^ This study provides the first systematic comparison of adsorption kinetics and mechanisms between monoelement and equimolar multicomponent systems, revealing condition-dependent rate-limiting steps. Combined with response surface optimization, this work establishes a mechanistically informed and practically relevant strategy for the recovery of Cu from CRM-rich waste streams.

## 2 Materials and Methods

### 2.1 Materials and Reagents

Only analytical-grade reagents were used to prepare the standard solutions. The salts of NdCl_3_·6H_2_O (358.69 g mol^–1^), DyCl_3_·6H_2_O (376.95 g mol^–1^), PrCl_3_·6H_2_O (247.27 g mol^–1^), GdCl_3_·6H_2_O (371.7 g mol^–1^), CoCl_2_·6H_2_O (237.93 g mol^–1^), and CuSO_4_·5H_2_O (249.68 g mol^–1^) were purchased from Sigma-Aldrich Riedel-de Haen. H_3_BO_3_ (61.83 g mol^–1^) was provided by the BDH Prolabo. NiCl_2_·6H_2_O (237.71 g mol^–1^) was acquired by Carlo Erba Reagents, and NaCl (58.44 g mol^–1^) was obtained from José Manuel Gomes dos Santos Lda. Graphenea provided the water dispersion of graphene oxide (0.4 wt %). The salinity of solutions was adjusted using an Eclipse model 45–63 hand-held refractometer. HNO_3_ 25%, v v^–1^ (Merck, Suprapur, 65%, m m^–1^), was used to wash all glass material for at least 24 h. After the acidic cleanup, they were cleaned with ultrapure water (Milli-Q water, 18 MΩ cm^–1^).

### 2.2 Thiourea-formaldehyde-Functionalized Graphene Oxide Synthesis

To synthesize the thiourea-formaldehyde (TF) resin, 0.04 mol of thiourea and 0.04 mol of formaldehyde (37% solution) were mixed, using magnetic stirring, in a round-bottomed glass. Under magnetic stirring, the solution was heated in an oil bath. The pH adjustment was performed using NaOH solution to reach between 7.8 and 9.0 and left under reflux at 60 °C for 30 min. Posteriorly, pH adjustment using an HCl solution was performed (pH = 4.5–5.5). The mixture was refluxed at 80 °C for at least 1 h. The end of the reaction was obtained with the appearance of a white solid at the bottom of the round-bottomed glass, which indicates the presence of the resin. After pH adjustment (pH = 7.0), the solution was cooled to room temperature. Water and ethanol were used to wash the solid, and it was dried at 30 °C. Later, 7.5 mL of GO solution (4 mg mL^–1^) was added to a round-bottom glass flask and subjected to ultrasonic treatment for 15 min. 7.5 mL of the TF resin was dissolved in dimethylformamide (DMF) solution (0.4 g mL^–1^), and it was added dropwise to the GO flask. The synthesized G3DTF powder was subjected to filtration and extensively washed with DMF and water to eliminate any excess resin. Subsequently, the purified material (G3DTF) was lyophilized for 24 h.

### 2.3 Thiourea-formaldehyde-Functionalized Graphene Oxide Characterization

The synthesized G3DTF material was characterized using multiple analytical techniques. Attenuated total reflectance Fourier transform infrared spectroscopy (ATR-FTIR) was performed on a Bruker Tensor 27 spectrometer (Bruker Corporation, USA) collecting spectra in the 400–4000 cm^–1^ region with 256 coadded scans and a spectral resolution of 4 cm^–1^ under controlled conditions (20 °C, 30% relative humidity). FT-Raman was performed on a Bruker Multiram using 500 scans at a resolution of 4 cm^–1^, over the range of 50–4000 cm^–1^, at a voltage intensity of 65 mW. Scanning electron microscopy (SEM) was carried out on a Hitachi TM4000 Plus microscope (Hitachi, Japan) at 15 kV to assess the surface morphology, with the sample mounted on a carbon-taped aluminum stub. Zeta potential measurements were conducted with a Malvern Panalytical Nano-ZS Zetasizer (Malvern, UK) to evaluate surface charge behavior as a function of pH. The specific surface area was determined by N_2_ adsorption isotherms employing the Brunauer–Emmett–Teller (BET) method using a Gemini Micromeritics system (Norcross, GA, USA). X-ray photoelectron spectroscopy (XPS) analyses took place using a SPECS Phoibos 150 hemispherical analyzer with delay-line detector and monochromatic Al Kα source (1486.74 eV) under ultrahigh vacuum (base pressure: 2 × 10^–10^ mbar). High-resolution C 1s, N 1s, and S 2p spectra were acquired at normal emission using a pass energy of 20 eV.

### 2.4 Sorption Assay

The sorption capacity of GO and G3DTF (graphene oxide, without and with functionalization, respectively, 1 g L^–1^) was evaluated in a mixture of B, Co, Cu, Ni, Pr, Nd, Gd, and Dy (100 μM of each element). The sorption assays were performed in 0.1 L Schott Duran flasks under magnetic stirring (350 rpm). Solutions were adjusted to pH 5.0 using a 0.01 M NaOH solution and held to pre-equilibrate for at least 24 h. Solution samples were collected in advance of material addition (0 h) and at stipulated times and stored in polystyrene tubes pretreated with HNO_3_ (65%, m m^–1^) to maintain pH < 2 at 4 °C until analysis. The assay was performed in duplicate, and quality control was followed by simultaneously running control solutions with the exposure solutions to assess potential losses of elements. The selected competitive ions (B, Co, Ni, Pr, Nd, Gd, and Dy) were chosen based on reported compositions of wind turbine permanent magnet leachates, ensuring a realistic assessment of Cu selectivity under CRM-rich conditions.

#### 2.4.1 Design of Experiments

The response surface methodology (RSM) is employed to evaluate the relationships between several quantitative factors and one or more responses, commonly expressed as an equation to optimize a given process.^30,31^ The Box–Behnken Design (BBD) was chosen as the experimental design, aligned with the RSM, to reduce the number of experimental runs without forfeiting model accuracy. Each variable was tested at three equidistant levels (−1, 0, 1), and the obtained full model was quadratic.^32^

A 3-factor–3-level BBD was used, considering sorbent dosage (0.2; 1.1; 2 g L^–1^), salinity (0; 15; 30 au), and pH (4; 6; 8 a.u.) as the independent factors and the removal efficiency of G3DTF (%, response) as the dependent factor. To evaluate the variability of the results, the central point was performed three times. This approach makes it possible to determine whether variations in responses are due to experimental error or the variables themselves, thereby increasing the reliability of the model. The BBD experiments are shown in Table 1.

**Table 1:** Coded and Actual Values Used for Each Experimental Trial^a^

	Coded values			Actual values		
Trial	X1	X2	X3	SD	S	P
1	–1	0	–1	0.2	15	4
2	+1	0	–1	2	15	4
3	–1	0	+1	0.2	15	8
4	+1	0	+1	2	15	8
5	0	–1	–1	1.1	0	4
6	0	+1	–1	1.1	30	4
7	0	–1	+1	1.1	0	8
8	0	+1	+1	1.1	30	8
9	–1	–1	0	0.2	0	6
10	–1	+1	0	0.2	30	6
11	+1	–1	0	2	0	6
12	+1	+1	0	2	30	6
13	0	0	0	1.1	15	6
14	0	0	0	1.1	15	6
15	0	0	0	1.1	15	6

a SD, sorbent dosage; S, salinity; P, pH.

Exposure conditions followed the methodology described in Section 2.4. When necessary, a pH adjustment was applied (4.0–8.0 with NaOH (0.1 M)), and the salinity was adjusted with NaCl. The experiments were performed under magnetic stirring for 24 h. Sample collection and storage were performed as described in Section 2.4.

Analysis of variance (ANOVA) verified the model’s response and the factors’ significance in the process. A second-order polynomial equation, presented in eq 1, correlated the factors with CRMs removal and predicted optimal conditions.^33^

$$Y=\beta_{0}+\overset{k}{\underset{i=1}{\sum }}\beta_{i}X_{i}+\overset{k}{\underset{i=1}{\sum }}\beta_{ii}X_{i}^{2}+\overset{k}{\underset{i<j}{\sum }}\beta_{ij}X_{i}X_{j}$$ 1

where *Y* is the predicted response value; β_0_ is a constant; β_*i*_, β_*ii*_, and β_*ij*_ are the linear coefficient, the quadratic coefficient, and the interaction coefficient, respectively; and *X* is the coded independent variable, which was calculated by eq 2.

$$X_{k}=\frac{x_{k}-x_{0}}{\Delta x_{k}}$$ 2

where *x*_*k*_ corresponds to the uncoded value of the independent variable; *x*_0_ denotes the value at the central point of the experimental domain; and Δ*x*_*k*_ denotes the spacing between levels of the *k* variable. The use of these coded variables facilitates the assessment of different factors on a standardized scale.

Response surface analyses utilized Design-Expert software (v.13.0.1.0, Stat-Ease, Inc., Minneapolis, USA). The coefficient of determination *R* ^2^ and adjusted coefficient *R*_Adj_^2^ (eqs 3 and 4, respectively) assessed the model’s goodness-of-fit, where *N*_DP_ represents experimental data points, *N*_P_ model parameters, *y*_*i*_ actual values, *ŷ*_*i*_ predicted values, and y̅ the experimental mean.

$$R^{2}=1-\frac{\sum {\left({ŷ}_{i}-y_{i}\right)}^{2}}{\sum {\left(y_{i}-\mathrm{y̅}\right)}^{2}}$$ 3

$$R_{Adj}^{2}=1-\left(1-R^{2}\right)\frac{\left(N_{DP}-1\right)}{\left(N_{DP}-N_{P}-1\right)}$$ 4

#### 2.4.2 Evaluation of a Solution Mimicking a Magnet Leachate

The removal efficiency of G3DTF was evaluated in a solution that mimics the concentrations of the main elements composing real magnet leachates (diluted 10-fold according to the composition in Peelman et al.,^34^ Table 2). The sorbent dosage of 1 g L^–1^ was used. Sorption testing, sampling, and storage were performed as stated in Section 2.4.

**Table 2:** Solution Mimicking the Concentrations of a Real Magnet Diluted 10-Fold

Element	B	Co	Cu	Ni	Pr	Nd	Gd	Dy
Concentration (mM)	5.1	2.5	0.15	0.05	0.32	7.7	0.03	1.8

### 2.5 Critical Raw Material Quantification

Concentrations of CRMs (B, Co, Cu, Ni, Pr, Nd, Gd, and Dy) in solution samples were measured by using Jobin Yvon *Activa M* inductively coupled plasma optical emission spectrometry. A multielement stock solution (IV-ICPMS 71A, Inorganic Ventures) diluted in 1% HNO_3_ served as the basis for calibration standards to quantify the elements. The limit of quantification was taken as the lowest point of the calibration curve (10 μg L^–1^), and calibration curves exhibiting correlation coefficients <0.999 were rejected.^35^

### 2.6 Kinetic Modeling and Sorption Isotherms

Kinetic adsorption models are used to monitor adsorption rates and provide insights into the underlying adsorption mechanisms. In this study, three nonlinear kinetic models were applied to describe the experimental data: the Lagergren pseudo-first-order model (PFO),^36^ Ho’s pseudo-second-order model (PSO),^37^ and the Elovich model.^38^

Sorption equilibrium data were studied using five isotherm models in their nonlinear forms: the two-parameter Langmuir,^39^ Freundlich,^40^ Dubinin–Radushkevich,^41^ and Temkin isotherm models^42^ and the three-parameter Sips, also known as the Langmuir–Freundlich isotherm model.^43^

Model fitting was achieved using GraphPad Prism 9 software,^44^ which was also used to perform all statistical analyses of the results (tests of significance). Equations of kinetic and isotherm models can be seen in the Supporting Information.

### 2.7 Desorption Assay

The viability of recovering Cu from G3DTF (post sorption assays in multielement solution) was assessed by immersing the Cu-enriched material in 150 mL of 10% v v^–1^ HNO_3_ under continuous stirring for 24 h and then analyzed by ICP-OES.

### 2.8 Data Analysis

The percentage of removal of Cu by G3DTF (Removal (%)), was obtained using the following equation:

$$\mathrm{Removal}\;\left(\%\right)=\frac{\left(C_{0}-C_{t}\right)}{C_{0}}\times 100$$ 5

where *C*_0_ (mg L^–1^) is the initial concentration of elements in solution and *C*_*t*_ (mg L^–1^) is the concentration of elements in solution at time *t*.

The concentration of Cu in G3DTF at a given period of time, *t*, *q*_*t*_ (mg g^–1^), was calculated using eq 6, assuming complete binding of the removed Cu to the material.

$$q_{t}\;\left(mg\;g^{-1}\right)=\frac{\left(C_{0}-C_{t}\right)\times V}{m}$$ 6

where *V* (L) is the volume of the solution, and *m* (g) is the mass of the material used in the experiment.

## 3 Results

### 3.1 Graphene Oxide and Thiourea-formaldehyde-Functionalized Graphene Oxide Sorption Studies

The capacity of G3DTF to remove Cu was initially evaluated in a monosolution of this element (Figure 1). A strong decline in *C*_*t*_/*C*_0_ in the first 30 min of contact (71%) can be observed, whereby the equilibrium state (98%) is reached after 3 h of contact. The variation in *C*_*t*_/*C*_0_ values in the control (solution without G3DTF) was <5% over time.

**Figure 1:**
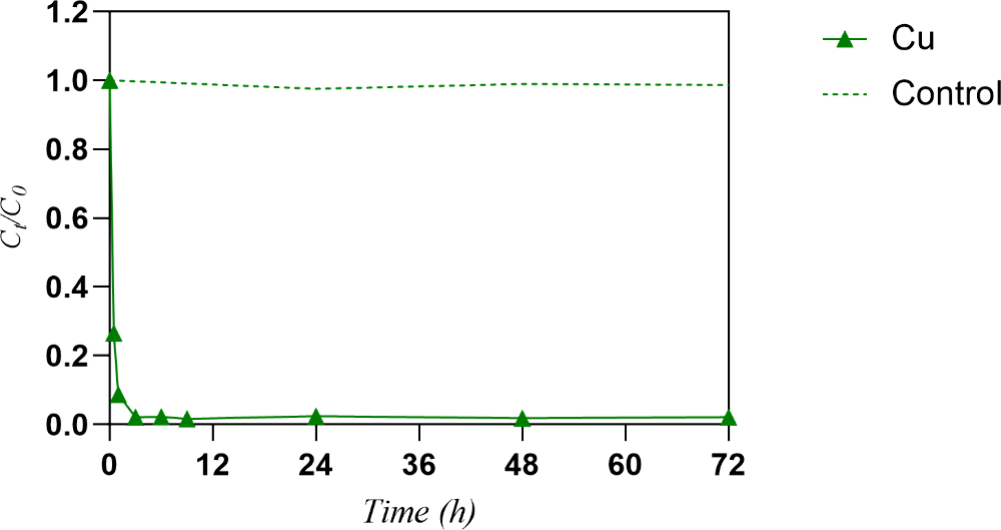
Variation of the normalized concentrations of Cu (*C*_t_/*C*_0_) in solution along time, in the absence and presence of G3DTF. Sorbent dosage of 1 g L^–1^, initial concentration of 100 μM, pH of 5, *n* = 2.

As G3DTF showed to be highly effective in removing Cu from a monosolution, its performance was evaluated in a multielement solution simulating a magnet leachate (100 μM of each element: B, Co, Cu, Ni, Pr, Nd, Gd, and Dy). All *C*_*t*_/*C*_0_ values in control (Figure S1) were within the defined intervals and did not change significantly over time (always less than 10%). Figure 2 shows the *C*_*t*_/*C*_0_ profiles of the CRMs in the equimolar multielement solution, in the presence of GO and functionalized GO (G3DTF). A strong decrease in the B *C*_*t*_/*C*_0_ was seen up to 6 h, in the presence of GO, pointing to a removal rate of over 60%. Until 3 h contact, the decrease in REE concentration followed the removal profile of B, but it was less accentuated in the following periods and reached an equilibrium state (50% removal). Cobalt, Ni, and Cu showed a slow removal profile in the first 6 h and reached equilibrium after 24 h (negligible removal of Co and Ni; Figure 2-I). On the other hand, Cu removal in the presence of G3DTF was marked up to 6 h (75%) and reached a steady-state after 24 h of contact with 99% removal. The remaining CRMs were negligibly removed even when the contact time was extended to 72 h, highlighting the selectivity of G3DTF toward Cu in the evaluated equimolar multielement solution.

**Figure 2:**
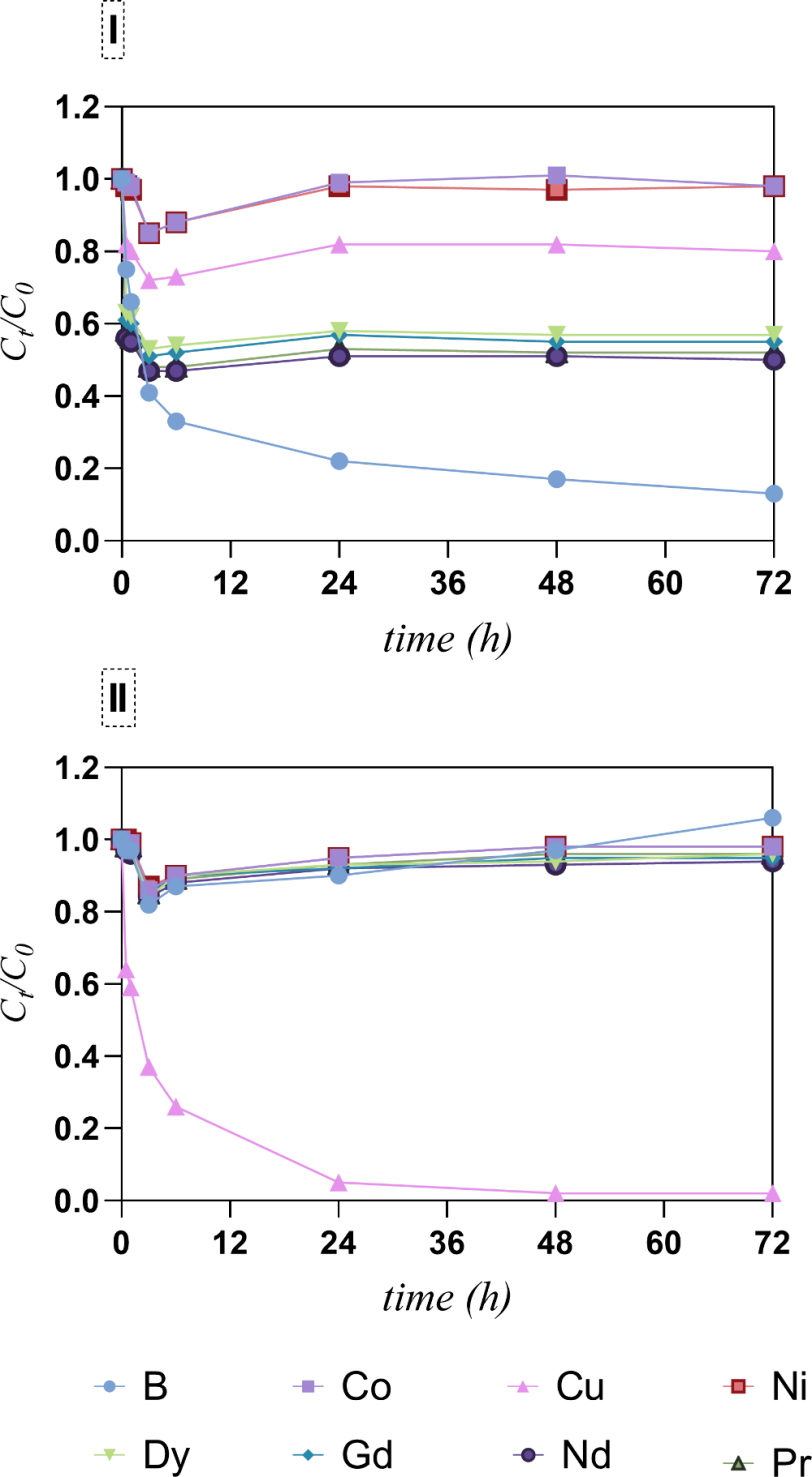
Normalized concentrations (*C*_t_/*C*_0_) of B, Co, Cu, Ni, Pr, Nd, Gd, and Dy as a function of contact time by (I) GO and (II) G3DTF. Sorbent dosage = 1 g L^–1^, initial concentration = 100 μM, pH = 5, *n* = 2.

### 3.2 Characterization of Thiourea-formaldehyde-Functionalized Graphene Oxide

Since G3DTF showed a high potential for the selective removal of Cu, the characterization of this material was carried out before and after contact with the equimolar multielement solution. Analysis of the FTIR spectra of G3DTF (Figure 3A) shows a similar pattern to that previously described by Gonçalves et al.^45^ The spectra of G3DTF, after multielement exposure, show the typical peaks at 3307 cm^–1^ (N–H stretching) and 3032 cm^–1^ (N–H of nonlinear H-bonded thiourea units’ deformation) of the TF polymer and the imine (C=N–H) peak at 1537 cm^–1^. The C–S bonds at 952 cm^–1^ and 661 cm^–1^ show a shift for higher wavenumber of 959 cm^–1^ and 657 cm^–1^. The polyether bridge indicated by the presence of the 1007 cm^–1^ (C–O–C) peak is still visible after exposure.^29^

**Figure 3:**
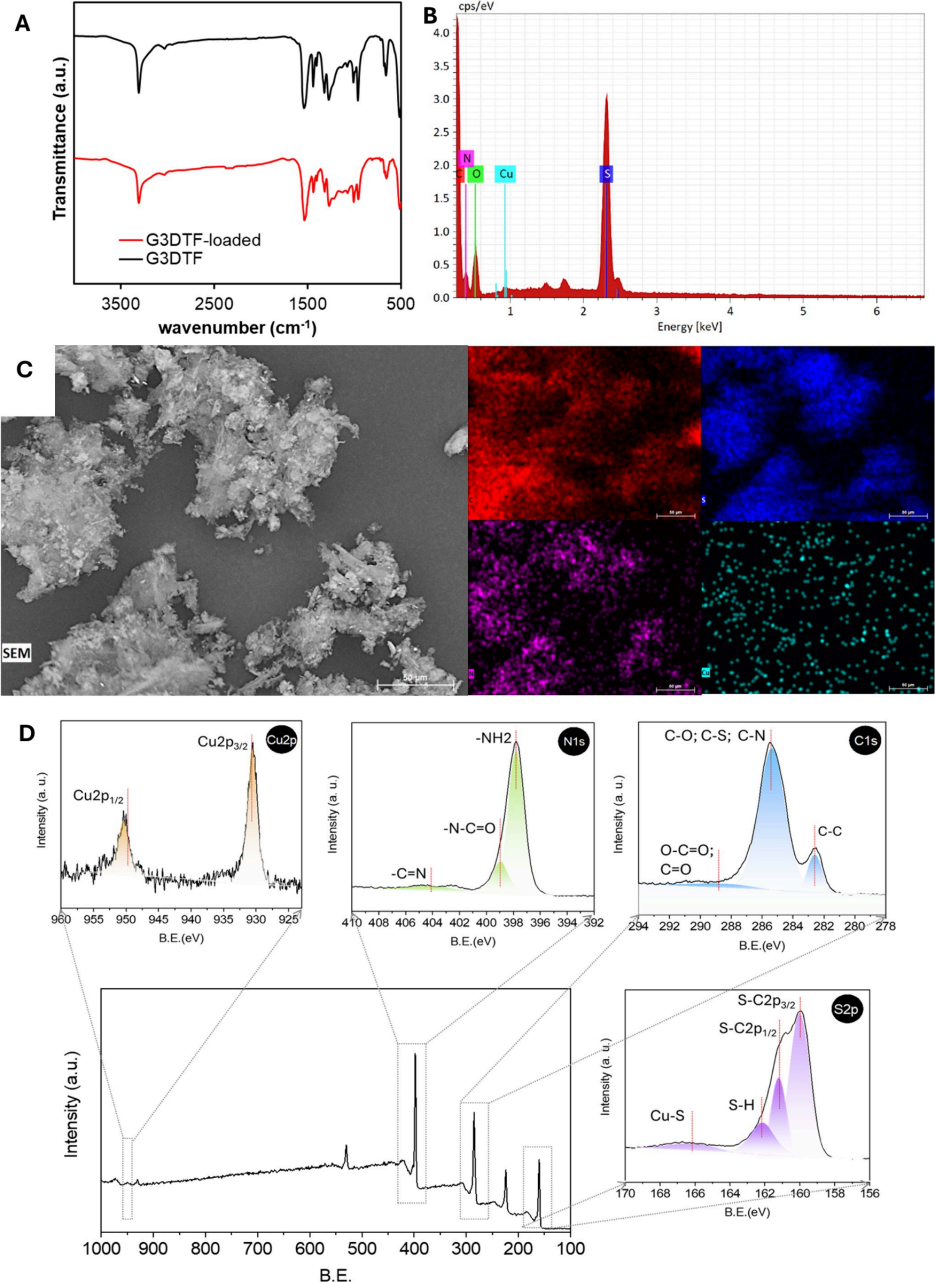
(A) FTIR spectra of G3DTF prior to and after multielement exposure; (B and C) SEM image of G3DTF powder along with the associated EDS mapping for Carbon (red), Sulfur (dark blue), Nitrogen (pink), and Copper (light blue) after exposure; and (D) XPS spectra for Cu 2p, N 1s, C 1s, and S 2p of G3DTF.

SEM-EDS examination of the powder after exposure (Figure 3B and C) revealed the presence of carbon, sulfur, and nitrogen, the typical elements of the thiourea structure (SC­(NH_2_)_2_), as well as the coexistence of Cu, confirming the interaction of the element with the surface of the G3DTF material.

High-resolution XPS analysis of G3DTF was conducted to investigate the binding mechanisms between the functionalized material and Cu (Figure 3D). The Cu 2p core-level spectrum exhibits two main peaks centered at approximately 932.8 and 952.6 eV, assigned to the Cu 2p_3/2_ and Cu 2p_1/2_ spin–orbit components, respectively, confirming the presence of Cu species in G3DTF (Figure 3D, Cu 2p). These binding energies are characteristic of Cu(0)/Cu­(I) species. In addition, the absence of Cu­(II) shakeup satellite features in the 941–945 eV region indicates that copper is predominantly present as Cu­(I)/Cu(0) rather than Cu­(II), consistent with previous reports for reduced or coordinatively stabilized Cu species.^46^

The C 1s core-level spectrum of G3DTF was deconvoluted into three main components. The dominant peak at 284.8 eV was assigned to C–C/C=C bonds from the GO structure (Figure 3D, C 1s).^45^ The higher-binding-energy component centered at approximately 287.6 eV was attributed to heteroatom-containing carbon species, including C–O, C–N, and C–S bonds, originating from GO nanosheets and the thiourea-based resin structure.^45^

The N 1s core-level spectrum was fitted with three components. The low-binding-energy peak at approximately 400 eV can be attributed to nitrogen species coordinated to Cu and/or deprotonated amine-like nitrogen, indicating strong electronic interaction between nitrogen atoms and Cu (Figure 3D, N 1s).^47^ Such low binding energies have been reported for metal–N coordination environments, where electron donation from nitrogen to the metal center and partial deprotonation result in increased electron density on nitrogen, leading to a downward shift of the N 1s binding energy into the 397–398 eV range.^47,48^ The second component at approximately 401.2 eV is assigned to −N–C=O, consistent with the covalent conjugation between the thiourea-derived polymeric network and GO. The third component at approximately 406.7 eV is attributed to oxidized or positively charged nitrogen species (e.g., protonated or quaternary nitrogen, −C=N^+^), which are commonly observed in oxidized or highly functionalized nitrogen-containing carbon materials.

The S 2p core-level spectrum exhibits multiple components associated with sulfur species in the thiourea-based resin (Figure 3D, S 2p), including S–C 2p_3/2_ (∼162.2 eV), S–C 2p_1/2_ (∼163.4 eV), S–H (∼164.9 eV), and oxidized sulfur species such as S=O (∼168.5 eV).^45^ In sulfur-containing polymer or ligand systems, Cu–S coordination is commonly reported in the 163–164 eV range, overlapping with S–C and S–H signals.^48^ Therefore, the S 2p spectrum may also include contributions from Cu–S coordination, and those interactions may coexist with the G3DTF structure.

The Raman spectrum (Figure S2) exhibits a prominent D band at approximately 1290 cm^–1^, attributed to defect-activated breathing modes of sp^2^ carbon rings, reflecting a high defect density within the structure and disruptions caused by oxidation in GO. The G band observed at approximately 1596 cm^–1^ corresponds to the in-plane vibration of sp^2^-bonded carbon atoms and reflects the presence of graphitic domains within the oxidized carbon network. In addition, second-order Raman features corresponding to the 2D band are observed in the range 2281–3020 cm^–1^, appearing broadened due to disorder and loss of long-range crystalline order, which is characteristic of GO.

### 3.3 Kinetic Modeling and Sorption Isotherm

Kinetic modeling of the experimental data for Cu sorption by G3DTF (1 g L^–1^), in both mono- and multielement solutions, is presented in Figure 4I and II and summarized in Table S1 (Supporting Information). Considering the monosolution, all kinetic models provided good fits, with PFO showing the best performance (*R* ^2^ = 0.999 and *S*_*y*,*x*_ = 18.75). Akaike’s Information Criterion (AIC)^49^ was used for model comparison and indicated a high probability that the PFO model is the correct one—99% against PSO or Elovich. The models also provided good fits in the multielement solution, with PFO performing slightly worse (*R* ^2^ = 0.933 and *S*_*y*,*x*_ = 633). PSO and Elovich models had better fits to the experimental data (0.982 < *R* ^2^ < 0.986), with low *S*_*y*,*x*_ values (330 and 294, respectively). Nevertheless, AIC indicates a higher probability of the Elovich model being the correct one (*R* ^2^ = 0.986 and *S*_*y*,*x*_ = 294)—99% against PSO or PFO.

**Figure 4:**
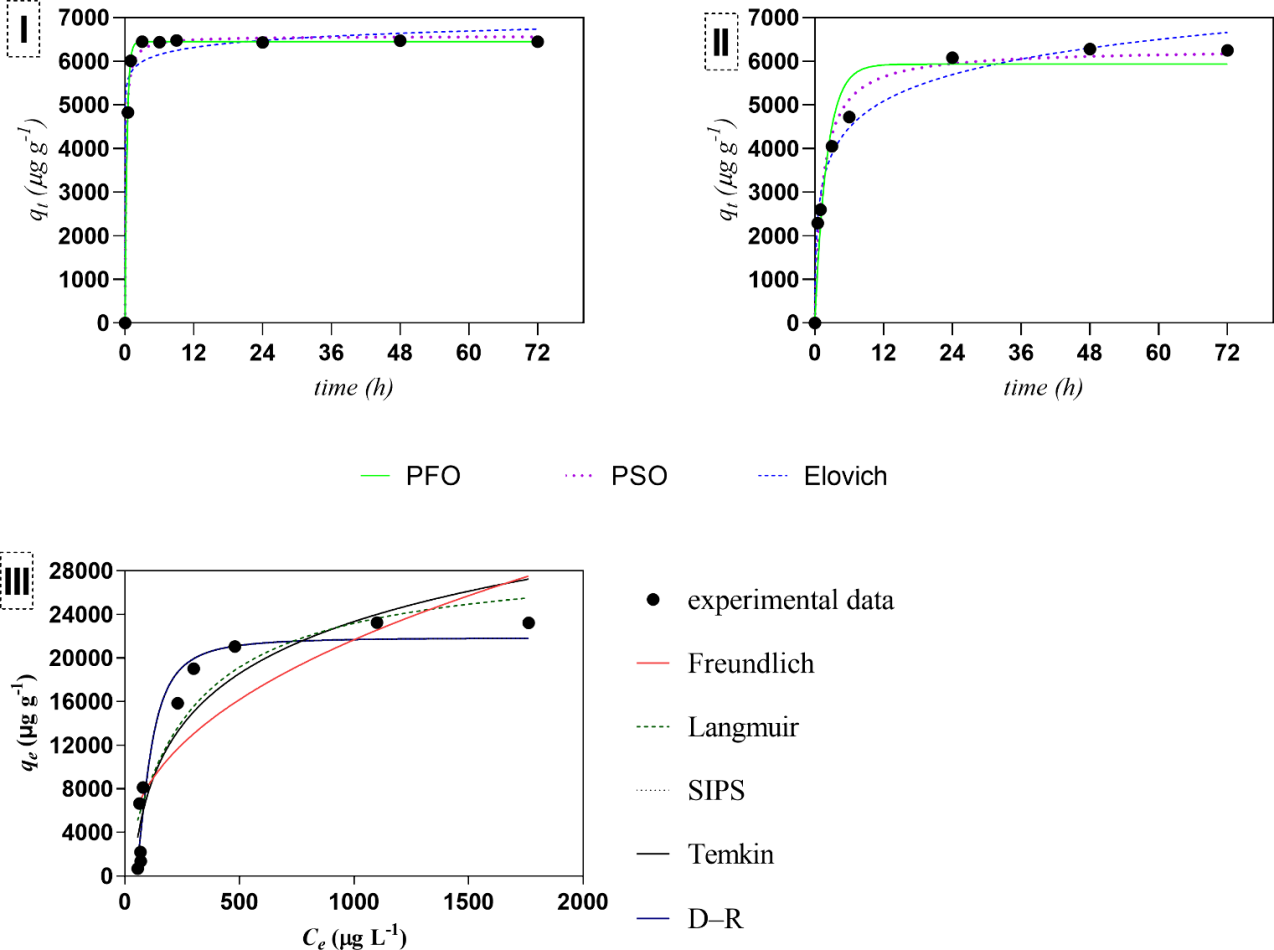
Kinetic modeling according to the PFO, PSO, and Elovich models, for the removal of (I) Cu in a monoelement solution and (II) Cu in a multielement system (B, Co, Cu, Dy, Gd, Nd, Ni, and Pr (100 μM)), using a dose of 1 g L^–1^ of G3DTF. (III) Isotherms (Freundlich, Langmuir, Temkin, Dubinin–Radushkevich (D–R), and SIPS models) for the Cu sorption onto G3DTF. Concentration of G3DTF in ultrapure water: 0.2, 0.25, 0.30, 0.35, 0.40, 0.50, 0.80, 1, 3, 5, 10 g L^–1^; and Cu of 100 μM.

The equilibrium data for Cu sorption onto G3DTF were fitted by using nonlinear isotherm models, and the resulting model fits are shown in Figure 4III. In Table S2 the goodness of fit and the parameters can be seen. The best fits for the experimental data were observed for the SIPS model (*R* ^2^ = 0.9556 and *S*_*y*,*x*_ = 2224) and the Dubinin–Radushkevich model (*R* ^2^ = 0.9464 and *S*_*y*,*x*_ = 2285), although the Dubinin–Radushkevich model has a higher probability of being the correct one, according to AIC = 88.7% against SIPS.

The free energy of adsorption, *E* (kJ mol^–1^), obtained using the Dubinin–Radushkevich model (*E* = 1/√2*B*) was calculated to be 19.1 kJ mol^–1^. The maximum Cu sorption capacity (*q*_max_), predicted by the SIPS isotherm model, ranged between 19.7 and 28.3 mg g^–1^.

### 3.4 Response Surface Models

The kinetic profile (Figure 2) showed that increasing the time from 24 to 72 h was not relevant to the sorption efficiency. Thus, 6 and 24 h were selected to proceed with response surface methodology (RSM) analysis and process optimization. Although the removal at some trials (data not shown) improved from 6 to 24 h, the fact that the removals of Cu in all the trials were very high and similar at 24 h made it impossible to adjust a model to the experimental data at this contact time. Thus, optimization was only performed for the contact time of 6 h. Results corresponding to the removal (%) at 6 h are presented in Table S3. For the multielement adsorption experiments, all metals were present at identical initial concentrations (100 μM each), ensuring that observed differences in removal efficiencies arise solely from variations in operational conditions.

The global between-replicate coefficients of variation (CV) of the BBD central point (factors found in their central values) were 0.05% for Cu, ensuring that the data were within normal dispersion and ensuring repeatability, thus validating the experimental design (Figure 5).

**Figure 5:**
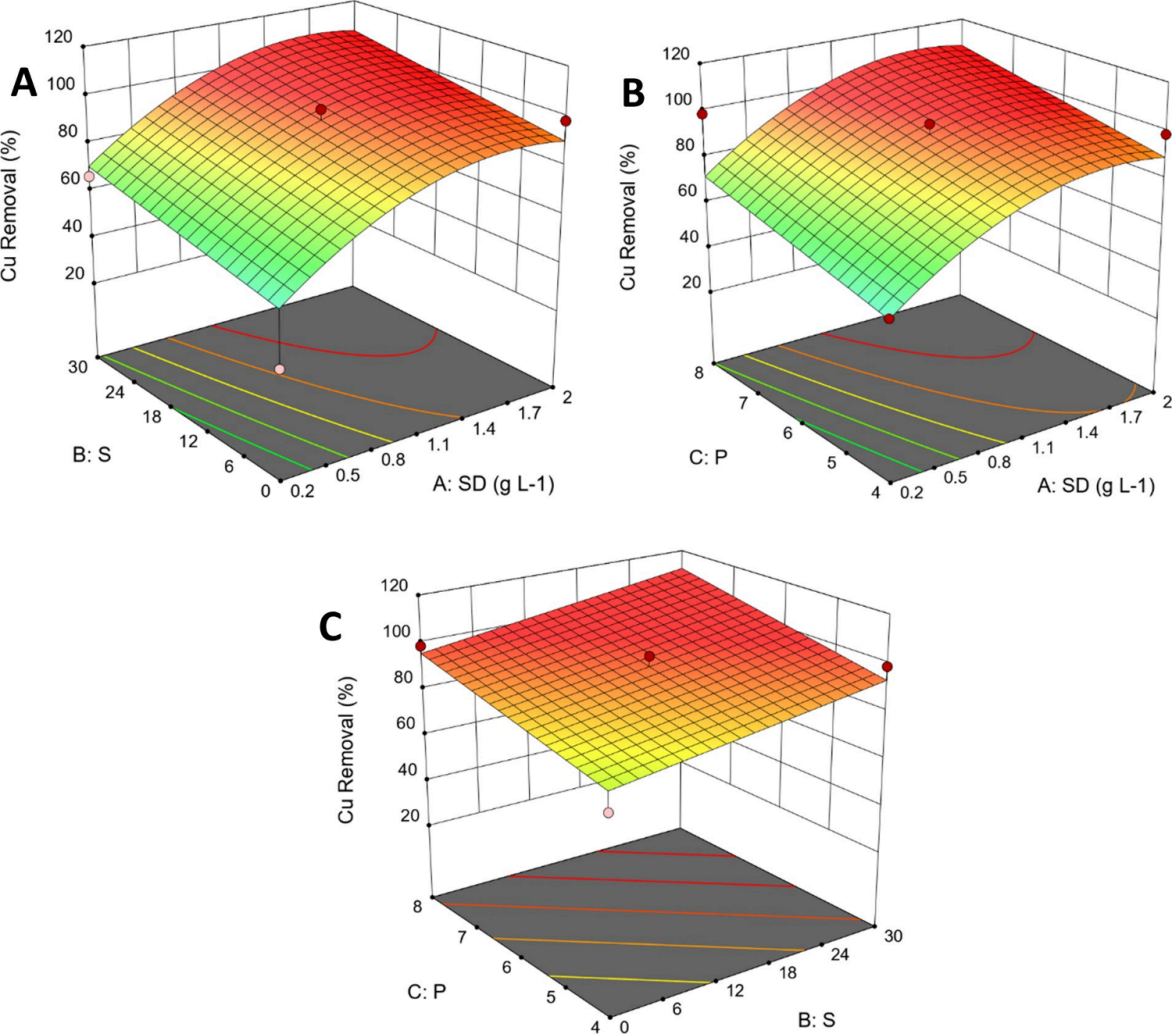
3D response surface for the removal of Cu by G3DTF, considering the three independent variables: (A) salinity (*S*) vs sorbent dosage (SD); (B) pH (P) vs SD; and (C) *P* vs *S* for 6 h of exposure.

To determine the statistically significant factors and their interactions influencing the removal process, analysis of variance (ANOVA, 95%) was conducted (Table S4) (bold values are significant (*p*-value <0.05) for the removal efficiency). Key findings regarding Cu removal using G3DTF show that sorbent dosage has the greatest impact on removal. The remaining factors were not significant for the removal of Cu from the studied multielement solutions. Although the sorbent dosage is crucial, its interactions with the remaining factors were not significant for the removal of Cu. Based on the obtained equation (*Y* = 14.9 + 57.9 × SD + 0.55 × *S* + 4.70 × *P* – 17.1 × SD^2^), 3D response surface plots were created and are shown in Figure 5. Regardless of pH and salinity, the minimum removal of Cu is 31% for a G3DTF dosage of 0.2 g L^–1^. An intermediate G3DTF amount of 1.1 g L^–1^ removed between 68 and 99% of Cu from the equimolar multielement solution.

Using the RSM, the models developed were optimized to maximize the removal of Cu while minimizing the removal of the other CRMs (Table 3). According to the predictions, it is possible to achieve virtually 100% Cu removal within 6 h of contact with G3DTF, while the removal of the other CRMs remains below 10%.

**Table 3:** Optimized Variable Values and Respective Removal (%) for CRMs at 6 h

		Optimal conditions			
	Element	Sorbent dosage	Salinity	pH	Removal %
6 h	B	1.1	Any	5.2	6.6
	Co				3.3
	Cu				100
	Ni				4.3
	Pr				3.5
	Nd				2.9
	Gd				2.0
	Dy				1.4

### 3.5 Removal of Copper from a Solution Simulating the Concentrations of a Real Magnet

To assess the selective recovery of Cu from real magnet leachate, G3DTF was evaluated in a solution that mimics the concentrations of the elements in real magnets^34^ (diluted 10-fold according to the composition - Table 4). The assay was performed using the optimized conditions determined by the BBD. The removal of Cu reached 93%, while the remaining elements in the solution were negligibly removed.

**Table 4:** Removal of CRMs by G3DTF from a Solution Simulating the Concentrations of a Real Magnet Were Diluted 10-Fold^a^

Elements	B	Co	Cu	Ni	Pr	Nd	Gd	Dy
Concentration (mM)	5.1	2.5	0.15	0.05	0.32	7.7	0.03	1.8
Removal (%)	2.83 ± 1.33	1.52 ± 0.02	93.5 ± 0.09	3.51 ± 0.09	0	4.33 ± 1.08	3.18 ± 0.07	1.96 ± 1.77

a Sorbent dosage: 1.1 g L^–1^, pH 5.2, salinity 0.

After sorption, the G3DTF material was applied in an acidic solution (HNO_3_ 10%) to assess the desorption of Cu. Results showed that after 24 h the solution was able to desorb 73 ± 1.9% of Cu from the material.

## 4 Discussion

Most studies approach Cu primarily as a potentially toxic element and therefore focus on its removal rather than recovery.^50^ Nonetheless, considering the ubiquity of this element in the industrial sector and its allocation on the most recent CRMs list, highlighting its importance as a raw material and its possible limitation regarding the supply (Chile is the main producer with 29%), alternative and sustainable approaches for its recovery are needed to guarantee the demand. Selective Cu removal from end-of life (EoL) magnets is therefore essential to simplifying processing and avoiding additional purification steps. Yet, there is still scarce information on the selective removal of Cu from multielement solutions and even from magnet leachates. The study addresses the true compositional complexity of wind turbine magnet leachate and the performance of thiourea-formaldehyde functionalized graphene oxide in the removal of Cu, at different levels of complexity (salinity, pH, and concentration range), evidencing the high selectivity of this material for Cu.

### 4.1 Thiourea-formaldehyde-Functionalized Graphene Oxide Synthesis

The synthesis of thiourea-formaldehyde-functionalized graphene oxide (G3DTF) can be understood through a two-step mechanism involving polymer formation, followed by surface functionalization of graphene oxide (GO).

In the first step, thiourea reacts with formaldehyde under controlled pH and temperature conditions, leading to a polycondensation reaction between the amine groups of thiourea and formaldehyde. This process results in the formation of methylene-bridged thiourea units (−NH–CH_2_–NH−), generating a TF polymer network enriched in sulfur and nitrogen functional groups.^51^

In the second step, the thiourea-formaldehyde polymer is immobilized onto graphene oxide sheets. The oxygen-rich surface chemistry on the GO sheets, for instance, epoxy, carboxyl, and hydroxyl groups, serves as an anchoring site for the polymer. Nucleophilic attack of thiourea amine groups on epoxy rings, together with hydrogen bonding and electrostatic interactions, promotes the immobilization of the polymer onto the GO surface.^52^ As a result, a hybrid material is obtained in which sulfur- and nitrogen-containing functional groups are homogeneously distributed across the GO framework.

The combined presence of sulfur and nitrogen donor atoms introduced by the TF polymer provides chemically tailored binding sites responsible for the strong and selective affinity of G3DTF for Cu ions.

### 4.2 Removal Efficiency

Control solution analysis (without G3DTF) showed a minimal variation of *C*_*t*_/*C*_0_ values, indicating that eventual decline of element concentration would not be associated with losses due to processes such as coprecipitation or adsorption onto the container walls, as well as potential contamination, which were negligible. Therefore, any observed decrease in *C*_*t*_/*C*_0_ values in the presence of the sorbent can be attributed primarily to interactions between the sorbent and the CRMs.

The curve corresponding to the removal of Cu by G3DTF is characterized by two different slope stages, in both mono- and multielement solutions. The first showed rapid removal, which is mostly linked to the pronounced gradient among the concentration of Cu in solution and that in G3DTF, where a diffusion process tends to occur.^44^ This phase corresponds to physicochemical adsorption governed by electrostatic interactions, ion exchange, chelation, and surface complexation mechanisms. In the subsequent stage, the adsorption rate declines and gradually reaches equilibrium, assigned to the progressive saturation of active sites on the surface of the material.^44^

According to Pearson,^53^ metal ions are classified into *hard*, *soft*, or intermediate metal ions and include information about simple ligands. Oxygen-donor ligands exhibit *hard* base character, while sulfur-donor ligands exhibit *soft* base character.^54^ In general, *hard* metals preferentially bind to *hard* ligands (carboxylate, carbonyl, alcohol, and phosphate), and the same is observed for *soft* metal ions and *soft* ligands (sulfydryl, thioether, and amino). However, in systems containing both hard and soft metal ions, the latter typically exhibit a higher affinity for coordinating ligands, often outcompeting and displacing essential hard metal ions from their binding sites.^54^ Essential metal ions (i.e., Na^+^, Mg^2+^, Ca^2+^) are considered *hard*, as rare earth elements, while Co^2+^, Ni^2+^ and Cu^2+^ are considered *borderline* metals. The monovalent Cu species (Cu^+^) is *soft*. In addition, the covalent index (CI) was created by Nieboer and McBryde (1973)^55^ and Nieboer and Richardson (1980)^56^ to classify metal ions using the expression *Xm* ^2^ *r* (*Xm* is the electronegativity while *r* is the ionic radius). Metal ions with higher covalency index (CI) values typically exhibit a greater tendency for covalent bond formation with ligands,^57^ which explains why the removal efficiency of G3DTF was higher for Cu (CI: Cu^+^ (3.47), Cu^2+^ (2.98), Co^2+^ (2.63), Ni^2+^(2.52), Dy^3+^ (1.36), Gd^3+^ (1.35), Nd^3+^ (1.29), and Pr^3+^ (1.29)).

Although functional groups rich in nitrogen and sulfur are recognized as key contributors to the removal of Cu in aqueous systems,^53,57^ the XPS analysis allowed us to observe and estimate the specific contribution of sulfur functional groups at BE of 166 eV. The Cu 2p_3/2_ peak position is characteristic of Cu bound to sulfur-containing ligands and is consistent with Cu^+^ species or strongly covalent Cu–S coordination environments. Importantly, the spectrum shows weak and poorly defined shakeup satellite features in the 940–945 eV region, which are typically intense for free or hydrated Cu^2+^ species. The attenuation of these satellite peaks suggests that Cu is predominantly stabilized through strong chemical interactions with sulfur donor atoms, rather than remaining as solvated Cu^2+^ ions. This observation supports the formation of Cu–S coordination bonds, in agreement with the S 2p signal at ∼166 eV assigned to Cu–S interactions.

### 4.3 Kinetic Modeling and Sorption Isotherm

In the monoelement solution, a fast sorption kinetics was observed, which was better described by PFO, suggesting that adsorption is governed solely by the intrinsic properties of the adsorbate.^58^

On the other hand, in contact with the multielement solution, the AIC pointed out that the Elovich model is most likely to describe the sorption mechanism of Cu removal by G3DTF, suggesting that chemisorption may be the limiting step (chemical bonds between the element and the G3DTF surface).^35^ This model relies on the assumption that the adsorption sites increase exponentially as adsorption proceeds, indicating a multilayer adsorption, whereby each layer has different activation energies for chemisorption.^59^

The sorption isotherms showed that the free energy (*E* > 16 kJ mol^–1^) of the adsorption supports that chemical forces dominate in the binding between Cu and G3DTF (chemisorption).^60^ According to the SIPS model, the heterogeneity index (*n*) of the surface energy was 0.4974, which indicates a moderately heterogeneous surface and suggests that the adsorption energy is not uniform across the surface.^61^ The adsorbate molecules preferentially occupy higher-energy sites first, followed by lower-energy sites as concentration increases.^60^

### 4.4 Copper Removal Mechanism

The selective adsorption of Cu by G3DTF is governed by different rate-limiting steps depending on the solution composition, reflecting the complexity of the adsorption process rather than a single universal mechanism. In monoelement Cu solutions, the adsorption is better represented by the PFO model, indicating that mass transfer and surface accessibility dominate the uptake rate under noncompetitive conditions.^62^ In this case, the abundance of available binding sites minimizes the influence of surface reaction limitations.

In contrast, in the multielement system, where Cu competes with base metals and REE, the Elovich model provides the best kinetic description. This behavior is characteristic of heterogeneous surfaces and adsorption processes controlled by surface chemical interactions.^63,64^ Under these competitive conditions, Cu adsorption becomes increasingly limited by the availability and reactivity of sulfur- and nitrogen-containing sites, consistent with a chemisorption-limited step.

The persistence of Cu uptake in high concentrations of competing ions indicates that adsorption is not controlled by electrostatic attraction or surface charge alone. Instead, Cu binding is dominated by specific coordination interactions that selectively stabilize Cu at sulfur- and nitrogen-rich sites. Competing ions, particularly REE, remain weakly interacting due to their preference for oxygen-donor environments and higher coordination requirements. This hierarchy of interactions explains why Cu adsorption remains favored under multicomponent conditions and clarifies the origin of the selectivity observed experimentally.

Taken together, these results indicate that while Cu binding to G3DTF involves strong chemical interactions, the overall adsorption mechanism is condition-dependent. Chemisorption via Cu–S–N coordination represents the primary binding interaction, whereas diffusion and site accessibility determine the observed kinetics depending on matrix complexity. This mechanistic distinction explains both the observed selectivity and the differences in kinetic behavior across experimental conditions.

### 4.5 Optimization

G3DTF was found to be a promising material for selective Cu removal, even in multielement solutions. Optimization studies for the removal of Cu from multielement solutions using G3DTF as a selective sorbent have not been reported in the literature to date. The DoE, based on the BBD approach, provided insights into the influence of various factors on sorption efficiency for the different elements in the mixture. Sorbent dosage was found to be the most significant factor (*p*-value < 0.05), while pH and salinity were not significant (*p*-value > 0.05). The increase in the sorbent dosage (more binding sites available) has a positive influence on the removal efficiency.^44^ Gonçalves et al.^45^ determined the point of zero charge (pzc) of G3DTF, and the material shows amphoteric behavior, with a pzc at pH ∼ 8.5, indicating that G3DTF has a positive charge at a pH < pzc and a negative charge at pH > pzc. Therefore, the sorption mechanism associated with the binding of Cu onto G3DTF is predominantly governed by intermolecular interactions, with covalent bonding playing a significant role. This chemical nature of the Cu–G3DTF interaction is further supported by the superior fit of the Elovich kinetic model, as previously discussed, indicating a chemisorption-dominated process.

### 4.6 Removal of Copper from a Solution Mimicking a Real Magnet Composition

Even though Cu was present in the solution, mimicking a real magnet composition at a concentration from 2 to 51 times lower, the material was still selective for Cu. G3DTF reached high removal efficiency for Cu when presented with a greater concentration of other ions, such as B, Co, Nd, Pr, and Dy. After sorption, Cu could be recovered from G3DTF using an acidic solution, proving a proof-of-concept for Cu recovery. This result shows the potential to selectively remove and recover Cu from wind turbine waste, minimizing mining extraction and promoting a circular economy. Nevertheless, desorption still requires further optimization in the future, and the long-term reusability of the material remains to be validated through multiple adsorption–desorption cycles.

The removal of Cu was already reported in the literature (Table 5). However, the majority of the studies were conducted using monoelement solutions. Only a few studies investigated the removal of Cu from multielement solutions containing only potentially toxic elements, for example, Pb, Hg, and Cd. Nevertheless, Cu removal from those mixtures (Pb, Hg, and Cd) was not selective.^65,66^ The maximum Cu adsorption capacity of G3DTF, determined in the present study, is 23.4 mg g^–1^ (SIPS isotherm), which is greater than those previously reported for alternative sorbents (except for GO-polydopamine, which shows a similar value, *q*_max_ = 24.4 mg g^–1^), even when those studies were carried out using unrealistically higher concentrations.

**Table 5:** Adsorption Parameters for Cu Removal Across Multiple Sorbents

Material	Sorbent dosage (g L^–1^)	pH	Salinity	Cu concentration (mg L^–1^)	Removal (%)	*q*_max_ (mg g^–1^)	ref.
GO-Polydopamine	-	5.2–6.8	-	50	-	24.4	^65^
GPBC-1	0.05	2–6	-	25–200	-	18.4	^67^
AC	0.1–0.4	5	-	100–900	-	20.2	^68^
Magnetite Nanoparticles	0.1	7	-	10	96	6.28	^69^
GO/Fe_3_O_4_	0.4	5.3	-	10	-	18.26	^70^
Magnetic GO	0.4	7	-	25	-	18.37	^66^
G3DTF	0.2–10	4.8	0	6.35	98	23.4	This study

The design of G3DTF resolves the fundamental limitation of poor selectivity that undermines most Cu sorbents in complex waste streams. By engineering sulfur- and nitrogen-rich coordination sites within a graphene oxide framework, G3DTF achieves intrinsic Cu selectivity under competitive conditions independent of electrostatic effects. This design further enables mechanistic differentiation between mono- and multicomponent systems and demonstrates direct applicability to wind-turbine-relevant leachates.

## 5 Conclusion

This study presents a novel thiourea-formaldehyde-functionalized graphene oxide (G3DTF) as a highly selective sorbent for Cu recovery from complex multielement systems relevant to EoL wind turbine magnets and other CRM-rich wastes. The incorporation of thiol and amine functional groups within the GO framework generated synergistic binding sites that exhibit an exceptional affinity for Cu, enabling nearly complete removal (≈99%) even in the presence of competing elements such as Co, Ni, and rare earth elements.

Kinetic and isotherm analyses, for the CRMs mixture, demonstrate that Cu adsorption involves strong chemical interactions with sulfur- and nitrogen-rich sites, with chemisorption becoming rate-limiting under competitive multielement conditions, supported by XPS evidence of Cu 2p and Cu–S bond formation involving specific coordination to thiourea sulfur sites, which explains the high selectivity observed in the presence of competing metal ions. Response surface methodology further demonstrated that sorbent dosage is the principal factor controlling removal efficiency, while pH and salinity exert negligible influence, underscoring the material’s robustness under varying environmental conditions. The optimized conditions (1.1 g L^–1^, pH 5.2) achieved maximal Cu recovery with minimal coextraction of other metals, validating the selectivity and process stability of G3DTF.

Notably, this work establishes the G3DTF as a new class of sulfur–nitrogen-functionalized graphene-based sorbent with unprecedented Cu selectivity in realistic multielement systems. Its strong performance in leachate-mimicking solutions highlights the potential of G3DTF to contribute to sustainable, circular recovery pathways for critical raw materials, reducing dependence on primary mining and supporting the objectives of the European Critical Raw Materials Act.

Future work should focus on scaling up synthesis, optimizing desorption efficiency for material reuse by conducting multicycle adsorption–desorption studies, and assessing long-term performance in real industrial waste streams.

## Supplementary Material



## Notes

Description of the kinetic and isotherm models, the normalized concentrations of the studied elements in solution during the control assay (Figure S1), the Raman spectra of G3DTF (Figure S2), the kinetic parameters of experimental data modeling referring to Cu removal, in a multielement system (Table S1), and the isotherm parameters of experimental data modeling referring to Cu (100 μM) removal (Table S2), the removal results obtained by the BBD model (Table S3), and the factors with the corresponding *p*-value evaluated by the BBD (Table S4) (PDF)
