# Supplementary material for: Thiourea-formaldehyde-Functionalized Graphene Oxide for the Selective Removal of Copper from Multielement Solution
Source: ACS Omega. 2026 Apr 1;11(14):22158–70. doi: 10.1021/acsomega.5c13435 (PMC13084448; doi:10.1021/acsomega.5c13435)
Supplement: Supplementary file 1 [file ao5c13435_si_001.pdf]

Supplementary material

A thiourea-formaldehyde functionalized graphene oxide for the selective removal of copper from multi-element solution

Nicole Ferreira[1,2*], Thainara Viana[1], Gil Gonçalves[3], Cláudia Nunes[4], Eduarda Pereira[1], Bruno Henriques[1]

[1]LAQV-REQUIMTE - Associated Laboratory for Green Chemistry, Department of Chemistry, University of Aveiro, 3810-193 Aveiro, Portugal
[2]CICECO - Aveiro Institute of Materials, Department of Chemistry, University of Aveiro, 3810-193 Aveiro, Portugal
[3]TEMA - Centre for Mechanical Technology and Automation, Mechanical Engineering Department, University of Aveiro, 3810-193 Aveiro, Portugal
[4]CICECO - Aveiro Institute of Materials, Department of Materials and Ceramics Engineering, University of Aveiro, 3810-193 Aveiro, Portugal

*Corresponding author. Number: +351 914793848, E-mail: nicoleviana@ua.pt

1.1. Kinect modelling and sorption isotherms

The adsorption kinetics study was carried out at different time intervals (from 0 to 72 h) with equimolar concentration using 1 g L^{-1} G3DTF. The equations of the kinetic models are as follows:

Lagergren pseudo-first-order model

$$q_t = q_e(1 - e^{k_1 t}) \quad \text{Eq. 1}$$

Ho's pseudo-second-order model

$$q_t = \frac{q_e^2 k_2 t}{1 + q_e k_2 t} \quad \text{Eq. 2}$$

Elovich model

$$q_t = \frac{1}{\beta}\, ln(1 + \alpha\beta t) \quad \text{Eq. 3}$$

where q_e (µg g^{-1}) is the amount of contaminant bound per unit of mass when equilibrium is reached, i.e., when there is no further variation in the concentration of contaminant in solution; k_1 is the rate constant of pseudo-first-order (h^{-1}); k_2 is

the rate constant of pseudo-second-order (g μg^{-1} h^{-1}); α is the initial sorption rate at zero coverage (μg g^{-1} h^{-1}) and β is the desorption constant (g μg^{-1}).

The sorption isotherm was carried out for 72 h, with equimolar concentration, using different dosages of G3DTF (from 0.2 to 10 g L^{-1}). The equations of the sorption isotherm models are as follows:

Langmuir model

$$q_e = \frac{q_m b_L C_e}{1 + b_L C_e} \quad \text{Eq. 4}$$

Freundlich model

$$q_e = K_F C_e^{1/n} \quad \text{Eq. 5}$$

Temkin model

$$q_e = \frac{RT}{b_T} lnK_T + \frac{RT}{b_T} lnC_e \quad \text{Eq. 6}$$

Dubinin–Radushkevich model

$$q_e = q_m \exp(-K\varepsilon^2) \quad \text{Eq. 7}$$

$$\varepsilon = RTln\left(1 + \frac{1}{C_e}\right) \quad \text{Eq. 8}$$

SIPS model

$$q_e = \frac{q_m (b_S C_e)^{1/n}}{1 + (b_S C_e)^{1/n}} \quad \text{Eq. 9}$$

where, q_m is the maximum sorption capacity (μg g^{-1}), b_L is the Langmuir constant related to the free energy of adsorption (L μg^{-1}), C_e is the concentration of metal sorbed in the equilibrium (μg L^{-1}), K_F is a constant related to the adsorption capacity of the sorbent (μg$^{1-1/n}$ L$^{1/n}$ g^{-1}) and n is the adsorption intensity or the heterogeneity of the sorbent, b_S is the Sips constant related to the energy of adsorption (L μg^{-1})$^{1/n}$, b_T is the Temkin constant related to the heat of sorption (kJ mol^{-1}), K_T is the equilibrium binding constant corresponding to the maximum binding energy (L g^{-1}), T is the absolute temperature (K), and R is the gas constant ($8.314 \times 10^{-3}\ kJ/mol\,K^{-1}$).

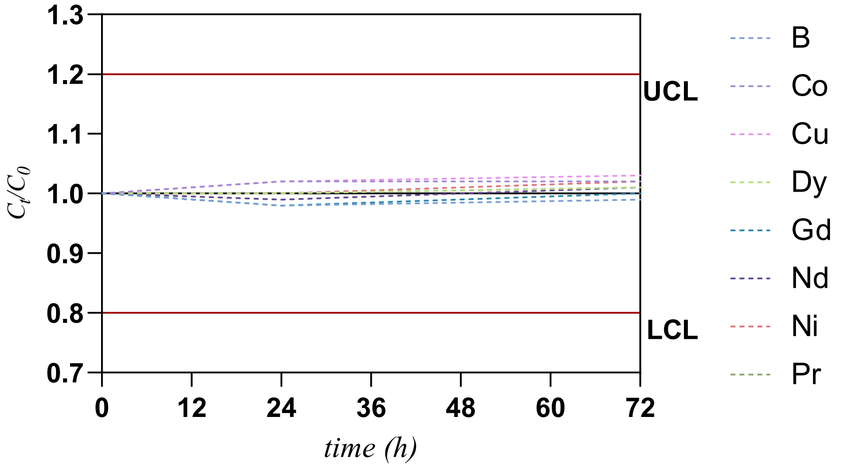

***Figure S1**: Normalised concentrations of the studied elements in solution during the control assay (control chart; UCL – Upper control limit; LCL – Lower control limit). Initial concentration of elements of 100 µM.*

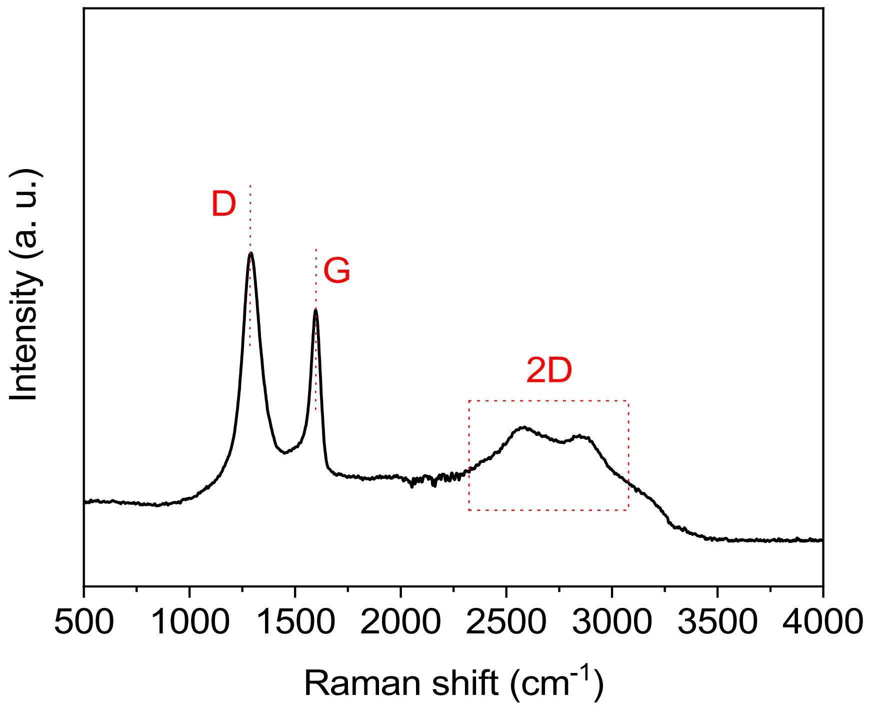

***Figure S2:** Raman spectra of G3DTF.*

Table S1: *Kinetic parameters of experimental data modelling referring to Cu removal, in a multi-element system, (B, Co, Cu, Dy, Gd, Nd, Ni, and Pr; 100 µM of each element), using a dose of 1 g L^{-1} of G3DTF. The PFO, PSO and Elovich models are considered. In bold are the best-fit data.*

Material	CRMs	Model	q_e (µg g^{-1})	k_1 ou k_2	β (g $µg^{-1}$)	α	R^2	$S_{y,x}$
G3DTF Mono-element	Cu	**PFO**	**6451**	**2.741**	**-**	**-**	**0.999**	**18.7**
		PSO	6573	$1.02×10^{-3}$	-	-	0.9943	172
		Elovich	-	-	$4.27×10^{-3}$	-	0.9700	395
G3DTF Multi-element	Cu	PFO	5933	0.47	-	-	0.933	633
		PSO	6285	$1.16×10^{-4}$	-	-	0.982	330
		**Elovich**	**-**	**-**	$\mathbf{1.14×10^{-3}}$	$\mathbf{2.36×10^{4}}$	**0.986**	**294**

Table S2: *Isotherm parameters of experimental data modelling referring to Cu (100 μM) removal, using a dose of 1 g L^{-1} of G3DTF. The Freundlich, Langmuir, Temkin, Dubinin-Radushkevich, and SIPS models are considered. In bold are the best-fit data.*

Model	Best fit values		Goodness of fit	
Freundlich	K_F (95% CI), μg g^{-1}	1176 (224-3893)	R^2	0.7597
	$1/n$ (95% CI)	0.4219 (0.2311-0.6597	$S_{y,x}$	4839
Langmuir	q_m (95% CI), μg g^{-1}	29375 (22313-339248)	R^2	0.8898
	b_L (95% CI), L μg^{-1}	3.75×10^{-3} (1.78×10^{-3}-7.63×10^{-3})	$S_{y,x}$	3277
Temkin	B (95% CI), J mol^{-1}	0.3602 (0.2803-0.5038)	R^2	0.8910
	K_t (95% CI), L μg^{-1}	2.97×10^{-2} (1.80×10^{-2}-6.69×10^{-2})	$S_{y,x}$	3258
**Dubinin–Radushkevich**	**q_m (95% CI), μg g^{-1}**	**21871 (19275-24505)**	**R^2**	**0.9464**
	**B (95% CI), mol^2/KJ^2**	**1.36×10^{-3} (9.70×10^{-4}-2.12×10^{-3})**	**$S_{y,x}$**	**2285**
SIPS	q_m (95% CI), μg g^{-1}	23409 (19693-28332)	R^2	0.9556
	$1/n$ (95% CI)	0.4974 (0.2283-0.8119)	$S_{y,x}$	2224
	b_S (95% CI)	6.63×10^{-3} (4.20×10^{-3}-1.06×10^{-2})		

In all experimental trials, the initial concentration of each element (B, Co, Cu, Dy, Gd, Nd, Ni, and Pr) in the multi-element solution was fixed at 100 µM. The Box–Behnken design was applied to vary operational parameters (three factors at three levels), not the solution composition. The variability in removal efficiencies, therefore, reflects differences in experimental conditions rather than differences in initial metal concentrations.

***Table S3**: Removal (%) of the elements from a multi-element solution (100 µM) at 6 h of exposure.*

	Removal (%)							
**Trial**	**B**	**Co**	**Cu**	**Dy**	**Gd**	**Nd**	**Ni**	**Pr**
**1**	5.60	8.07	53.2	5.69	3.87	5.95	5.86	6.18
**2**	13.1	12.4	98.7	13.2	12.0	14.0	13.2	14.6
**3**	18.5	27.1	98.6	87.9	84.3	77.1	30.0	72.3
**4**	16.8	16.8	98.6	6.95	7.50	7.74	17.1	10.2
**5**	11.1	11.8	68.4	10.9	10.7	10.6	10.1	10.8
**6**	6.90	8.98	98.7	10.0	11.0	9.29	10.1	11.0
**7**	13.6	16.4	98.6	84.0	80.7	77.2	19.8	74.5
**8**	4.20	9.35	98.4	75.2	67.9	57.1	10.3	52.1
**9**	7.20	5.93	30.9	4.02	6.37	6.76	6.17	6.79
**10**	8.60	0.00	66.2	0.64	1.66	0.68	0.34	0.66
**11**	8.90	4.63	98.4	5.44	5.98	7.08	3.24	5.03
**12**	8.20	0.87	98.7	3.97	1.87	3.17	5.31	5.25
**13**	17.8	20.2	98.6	18.2	19.0	19.1	18.0	17.1
**14**	13.9	12.7	98.6	12.0	12.4	12.8	10.5	12.2
**15**	7.50	6.19	98.7	7.57	7.64	5.73	5.57	9.20

***Table S4**: Factors with the corresponding p-value for a contact time of 6 h. Bold values are significant (p-value < 0.05) for the removal efficiency.*

	Coefficients (*p*-value)								
**Element**	SD	S	P	SD-S	SD-P	S-P	SD^2	S^2	P^2
**B**	0.6260	0.3833	0.2832	0.8362	0.3874	0.6132	0.9617	0.1220	0.8230
**Co**	0.6916	0.2550	0.1220	0.8483	0.2317	0.7133	0.3548	**0.0476**	0.0883
**Cu**	**0.0041**	0.1257	0.0847	-	-	-	0.0826	-	-
**Ni**	0.8189	0.4005	**0.0471**	0.4760	0.1050	0.4000	0.5280	0.0814	**0.0472**
**Pr**	0.1471	-	**0.0004**	-	**0.0116**	-	-	-	**0.0027**
**Nd**	0.1282	-	**0.0005**	-	**0.0112**	-	-	-	**0.0036**
**Gd**	0.0920	-	**0.0003**	-	**0.0087**	-	-	-	**0.0026**
**Dy**	0.1208	-	**0.0004**	-	**0.0115**	-	-	-	**0.0027**
